# Healthcare utilisation of 282,080 individuals with long COVID over two years: a multiple matched control, longitudinal cohort analysis

**DOI:** 10.1177/01410768241288345

**Published:** 2024-11-27

**Authors:** Yi Mu, Ashkan Dashtban, Mehrdad A Mizani, Chris Tomlinson, Mohamed Mohamed, Mark Ashworth, Mamas Mamas, Rouven Priedon, Steffen Petersen, Evan Kontopantelis, Kim Horstmanshof, Christina Pagel, Mevhibe Hocaoğlu, Kamlesh Khunti, Richard Williams, Johan Thygesen, Paula Lorgelly, Manuel Gomes, Melissa Heightman, Amitava Banerjee, on behalf of the CVD-COVID-UK/COVID-IMPACT Consortium

**Affiliations:** 1 Institute of Health Informatics, 4919 University College London, London, UK; 2 British Heart Foundation Data Science Centre, Health Data Research UK, London, UK; 3 Cardiology, Barts Health NHS Trust, London, UK; 4 School of Population Health and Environmental Sciences, King’s College London, London, UK; 5 National Institute for Health Research School for Primary Care Research, Division of Population Health, Health Services Research and Primary Care, School of Health Sciences, Faculty of Biology, Medicine and Health, Manchester Academic Health Science Centre, University of Manchester, Manchester, UK; 6 Keele Cardiovascular Research Group, Centre for Prognosis Research, Keele University, Keele, UK; 7 Department of Cardiology, Royal Stoke University Hospital, Stoke-on-Trent, UK; 8 William Harvey Research Institute, NIHR Biomedical Research Centre at Barts, Queen Mary University of London, London, UK; 9 Division of Informatics, Imaging and Data Science, Faculty of Biology, Medicine and Health, Manchester Academic Health Science Centre, University of Manchester, Manchester, UK; 10 Clinical Operational Research Unit, University College London, London, UK; 11 Cicely Saunders Institute of Palliative Care, Policy & Rehabilitation, King’s College London, London, UK; 12 Diabetes Research Centre, University of Leicester, Leicester, UK; 13 Division of Informatics, Imaging and Data Science, Faculty of Biology, Medicine and Health, Manchester Academic Health Science Centre, University of Manchester, Manchester, UK; 14 NIHR Applied Research Collaboration Greater Manchester, The University of Manchester, Manchester, UK; 15 School of Population Health, Health Systems and School of Business, University of Auckland, Auckland, New Zealand; 16 Institute of Epidemiology and Health, University College London, London, UK; 17 University College London Hospitals NHS Foundation Trust, London, UK; 18 Health Data Research UK, University College London, London, UK

**Keywords:** Epidemiology, health economics, health policy, public health

## Abstract

**Objectives:**

To investigate healthcare utilisation and cost in individuals with long COVID (LC) at population level.

**Design:**

Case–control cohort analysis with multiple age-, sex-, ethnicity-, deprivation-, region- and comorbidity-matched control groups: (1) COVID only, no LC; (2) pre-pandemic; (3) contemporary non-COVID; and (4) pre-LC (self-controlled, pre-COVID pandemic).

**Setting:**

National, population-based, linked UK electronic health records (British Heart Foundation/NHS England Secure Data Environment).

**Participants:**

Adults aged ≥18 years with LC between January 2020 and January 2023.

**Main outcome measures:**

Healthcare utilisation (number of consultations/visits per person: primary care (general practitioner [GP]), secondary care (outpatient [OP], inpatient [IP] and emergency department [ED], investigations and procedures) and inflation-adjusted cost (£) for LC and control populations per month, calendar year and pandemic year for each category.

**Results:**

A total of 282,080 individuals with LC were included between January 2020 and January 2023. The control groups were COVID only, no LC (*n* = 1,112,370), pre-pandemic (*n* = 1,031,285), contemporary non-COVID (*n* = 1,118,360) and pre-LC (*n* = 282,080). Healthcare utilisation per person (per month/year) was higher in LC than controls across GP, OP and ED. For IP, LC had higher healthcare utilisation than pre-LC and contemporary non-COVID (all *p* < 0.0001). Healthcare utilisation of the LC group increased progressively between 2020 and 2023, compared with controls. Median cost per patient/year was also higher in individuals with LC than all control groups.

**Conclusions:**

LC has been associated with substantial, persistent healthcare utilisation and cost over the last three years. Future funding, resources and staff for LC prevention, treatment and research must be prioritised to reduce sustained primary and secondary healthcare utilisation and costs.

## Introduction

In the UK alone, long COVID (LC), defined as persistent symptoms following acute COVID beyond four weeks, currently affects 1.9 million individuals.^1^ Following coronavirus disease 2019 (COVID-19), 45% of individuals, regardless of hospitalisation status, have symptoms at four months.^2^ In US veterans, at two years, 31% of non-hospitalised and 65% of hospitalised individuals with COVID-19 had LC.^3^ At least 65 million individuals worldwide are estimated to have had LC in the first pandemic wave.^4^ Despite scientific progress, this new, complex syndrome is still not fully defined, whether by pathophysiology, epidemiology, prevailing subtypes or treatments.^4–7^ Health professionals, researchers, policymakers and patients are following a learning health system approach, treating at the same time as deciphering underlying mechanisms.^8^

LC is a heterogeneous, complex condition affecting multiple organs, probably by multiple underlying mechanisms and trajectories.^3,9^ For example, at six months, one in five individuals with LC had cardiac abnormalities on MRI (left or right ventricular dysfunction or dilatation), persisting in over half of those at 12 months.^10^ Single- and multi-organ abnormalities were present in 69% and 23% at six months, persisting in 59% and 27% at one-year follow-up (FU), respectively.^9^ Different organ abnormality patterns necessitate different clinical specialties, investigations and potentially different levels of care, monitoring, FU, resourcing and costs.^3,11^

Health systems have been under strain from direct, indirect and long-term COVID-19 effects and resources have been constrained in LC service provision. NHS England uniquely rolled out a specialist LC clinical service from late 2020, consisting of 100 dedicated clinics.^12,13^ Health system burden is major, and even in a high-income setting, staff and infrastructure have been stretched. For better understanding of current and future LC healthcare needs, healthcare utilisation and costs at scale need to be quantified for budgetary, service and policy planning.^14^ However, these are poorly studied and characterised to-date, with limited studies in few countries, including Germany, Israel and USA.^15–17^ Contemporary studies have excluded relevant control groups (e.g. contemporary and pre-pandemic non-COVID-19 populations), which may enable clearer understanding of healthcare use.^18^

Using national, linked electronic health records (EHRs), we conducted a population-based analysis of healthcare utilisation and cost in individuals with LC, comparing with multiple control populations before and during the COVID-19 pandemic.

## Methods

### Study population

We identified individuals ≥18 years of age with diagnosis of LC between 1 January 2020 until 31 January 2023, based on validated EHR phenotypes^19^ in the CVD-COVID-UK/COVID-IMPACT consortium (https://bhfdatasciencecentre.org/areas/cvd-covid-uk-covid-impact/), using data accessed in NHS England’s Secure Data Environment (SDE) service for England, via the British Heart Foundation Data Science Centre’s CVD-COVID-UK/COVID-IMPACT consortium. The LC phenotypes relied on 20 SNOMED-CT and one ICD-10 codes in primary and secondary care datasets, respectively (Table S1), including COVID General Practice Extraction Service Data for Pandemic Planning and Research, Hospital Episode Statics (including outpatient [OP] and Admitted Patient Care and Emergency Care Data Set). As well as diagnostic codes, we included referral/assessment codes, to identify as many individuals with LC as possible, since clinical LC coding was generally under-utilised.

### Control populations

We identified three matched control populations: (1) ‘COVID only, no LC’, including people who had COVID-19 but never developed LC (i.e. no LC and diagnostic codes of COVID-19); (2) ‘Pre-Pandemic’, including people prior to 31 December 2019, unaffected by pandemic health and healthcare disruptions; and (3) ‘Contemporary Non-COVID’, as a no COVID-19 (defined by no positive test in Second Generation Surveillance System or SGSS and no diagnostic code) and no LC primary care codes in individuals who never had COVID-19 until 1 April 2022 (after which COVID-19 testing was no longer freely available in England). We included a fourth self-control population: (4) ‘Pre-LC group’, including pre-pandemic data for individuals who went on to develop LC (Table 1).

**Table 1.** Definitions of study and matched control populations for analysis of healthcare utilisation in long COVID.

Population cohort	Definition	Study period	Index date	Match	Matching success rate
Long COVID (LC)	Long COVID (study population)	First LC diagnosis until 31 January 2023	28 Jan 2020	N/A	N/A
Pre-long COVID (Pre-LC)	Pre-pandemic medical history in those later developing long COVID (same as study population)	1 Jan 2018–31 Dec 2019	31 Dec 2017	N/A	N/A
COVID-19 only, no long COVID (COVID-19 only, no LC)	COVID-19 but no development of long COVID	COVID-19 between 29 Jan 2020 and 1 Apr 2022, and followed up until 31 January 2023	28 Jan 2020	Age, sex, ethnicity, IMD, region, comorbidities	98.5%–99.4%
Pre-pandemic	Prior to the pandemic, i.e. no SARS-CoV-2 infection	1 Jan 2018–31 Dec 2019	31 Dec 2017	Age, sex, ethnicity, IMD, region, comorbidities	87.2%–97.5%
Contemporary non-COVID-19	No SARS-CoV-2 infection or long COVID	29 Jan 2020–1 April 2022	28 Jan 2020	Age, sex, ethnicity, IMD, region, comorbidities	98.7%–99.6%

COVID-19: coronavirus disease 2019; SARS-CoV-2: severe acute respiratory syndrome coronavirus 2; IMD: Index of Multiple Deprivation.

Each matched control population was exactly matched without replacement (1:4 ratio over four consecutive rounds) for the following variables: age (seven 10-year interval bands: 18–19, 20–29, 30–39, 40–49, 50–59, 60–69, >70), sex (male or female), ethnicity (being white or non-white), Index of Multiple Deprivation (IMD) quintiles (1–5, 1: greatest socioeconomic deprivation, 5: least), geographical regions (East of England, London, Midlands, North East and Yorkshire, North West, South East, South West) and seven pre-existing co-morbidities (cardiovascular disease (CVD), hypertension, diabetes, Chronic Obstructive Pulmonary Disease (COPD), asthma, depression, cancer). We found and assigned distinct controls to each case. Each case had four rows in the final table with four distinct controls, making numbers of cases and controls the same (1:1). The Pre-LC group included the same individuals as the study population, removing need for matching (Table 1). Some control groups may overlap. For example, the Pre-Pandemic group was extracted based on the pre-pandemic cohort, including almost the entire England population alive on 1 January 2018. Therefore, the overlap between the Pre-Pandemic group and other control groups was natural, but very low (1%–8%). Some control groups were, by definition, mutually exclusive. For example, Pre-LC group and Contemporary Non-COVID had no overlap (Table S3).

### Healthcare utilisation

For the study and control populations, we assessed healthcare utilisation per month and per year, including general practitioner (GP) consultations, OP appointments, inpatient (IP) admissions (including number, duration in general ward and critical care) and emergency department (ED) attendances, by dividing total healthcare utilisation in each category (GP, OP, IP, ED) for each patient by FU periods (in months/years). For example, a patient with nine months FU would have nine months and 0.75 years as denominators for the estimation. If multiple same-day records were found, it was counted as one consultation, admission or attendance, as appropriate. Prescription records were not included in analyses. For each population, we estimated median utilisation in each category (GP, OP, IP, ED), comparing between LC and each control group, using one-sided Mann-Whitney U test with null hypotheses that healthcare utilisation in the corresponding department (GP/OP/IP/CC/ED) for LC is less than for controls (Table 3).

**Table 3.** Healthcare utilisation and all-cause mortality for individuals with long COVID, compared with control populations.

		LC	Pre-LC	COVID-19 only, no LC	Pre-pandemic	Contemporary non-COVID-19
	*N*	282,080	282,080	1,112,370	1,031,285	1,118,360
	Follow-up (days)	395 (177)	730	495 (187)	730 (6)	780 (70)
	Mean (SD)	range: 0–1125		range: 0.00–1096	range: 3–730	range: 1–791
Per month						
GP consultation	N^a^	0.82 [0.46 1.42]	0.25 [0.08 0.54]	0.59 [0.31 1.09]	0.29 [0.08 0.63]	0.46 [0.23 0.88]
	Excess^b^		−0.57, *p* < 2.2 × 10^−16^	−0.23, *p* < 2.2 × 10^−16^	−0.53, *p* < 2.2 × 10^−16^	−0.36, *p* < 2.2 × 10^−16^
Outpatient appointment	N	0.09 [0.00 0.35]	0.04 [0.00 0.21]	0.00 [0.00 0.18]	0.04 [0.00 0.25]	0.04 [0.00 0.15]
	Excess		−0.05, *p* < 2.2 × 10^−16^	−0.09, *p* < 2.2 × 10^−16^	−0.05, *p* < 2.2 × 10^−16^	−0.05, *p* < 2.2 × 10^−16^
Inpatient admission	N	0.00 [0.00 0.05]	0.00 [0.00 0.04]	0.00 [0.00 0.00]	0.00 [0.00 0.04]	0.00 [0.00 0.04]
	Excess		0.00, *p* < 2.2 × 10^−16^	0.00, *p* < 2.2 × 10^−16^	0.00, *p* = 0.2111	0.00, *p* < 2.2 × 10^−16^
	Duration^c^	0.00 [0.00 0.00]	0.00 [0.00 0.00]	0.00 [0.00 0.00]	0.00 [0.00 0.00]	0.00 [0.00 0.00]
	Excess		0.00, *p* = 0.9997	0.00, *p* = 1	0.00, *p* = 1	0.00, *p* = 0.2257
Critical care	Duration	0.00 [0.00 0.00]	0.00 [0.00 0.00]	0.00 [0.00 0.00]	0.00 [0.00 0.00]	0.00 [0.00 0.00]
	Excess		0.00, *p* < 2.2 × 10^−16^	0.00, *p* = 1	0.00, *p* = 1	0.00, *p* = 9.96 × 10^−11^
ED attendance	N	0.00 [0.00 0.07]	0.00 [0.00 0.04]	0.00 [0.00 0.06]	0.00 [0.00 0.00]	0.00 [0.00 0.04]
	Excess		0.00, *p* < 2.2 × 10^−16^	0.00, *p* < 2.2 × 10^−16^	0.00, *p* < 2.2 × 10^−16^	0.00, *p* < 2.2 × 10^−16^
Cost (£)^d^		58.74 [25.24 129.99]	24.47 [7.10 66.73]	37.27 [15.45 89.10]	25.47 [5.68 71.78]	29.13 [11.76 72.79]
	Excess		−34.27, *p* < 2.2 × 10^−16^	−21.47, *p* < 2.2 × 10^−16^	−33.27, *p* < 2.2 × 10^−16^	−29.61, *p* < 2.2 × 10^−16^
Per year						
GP consultation	N	9.90 [5.47 16.99]	3.01 [1.00 6.51]	7.07 [3.73 13.05]	3.51 [1.00 7.52]	5.53 [2.77 10.61]
	Excess		−6.89, *p* < 2.2 × 10^−16^	−2.83, *p* < 2.2 × 10^−16^	−6.39, *p* < 2.2 × 10^−16^	−4.37, *p* < 2.2 × 10^−16^
Outpatient appointment	N	1.07 [0.00 4.15]	0.50 [0.00 2.51]	0.00 [0.00 2.22]	0.51 [0.00 3.01]	0.46 [0.00 1.84]
	Excess		−0.57, *p* < 2.2 × 10^−16^	−1.07, *p* < 2.2 × 10^−16^	−0.56, *p* < 2.2 × 10^−16^	−0.61, *p* < 2.2 × 10^−16^
Inpatient admission	N	0.00 [0.00 0.64]	0.00 [0.00 0.50]	0.00 [0.00 0.00]	0.00 [0.00 0.50]	0.00 [0.00 0.46]
	Excess		0.00, *p* < 2.2 × 10^−16^	0.00, *p* < 2.2 × 10^−16^	0.00, *p* = 0.2113	0.00, *p* < 2.2 × 10^−16^
	Duration	0.00 [0.00 0.00]	0.00 [0.00 0.00]	0.00 [0.00 0.00]	0.00 [0.00 0.00]	0.00 [0.00 0.00]
	Excess		0.00, *p* = 0.9997	0.00, *p* = 1	0.00, *p* = 1	0.00, *p* = 0.2244
Critical care	Duration	0.00 [0.00 0.00]	0.00 [0.00 0.00]	0.00 [0.00 0.00]	0.00 [0.00 0.00]	0.00 [0.00 0.00]
	Excess		0.00, *p* < 2.2 × 10^−16^	0.00, *p* = 1	0.00, *p* = 1	0.00, *p* = 9.96 × 10^−11^
ED attendance	N	0.00 [0.00 0.82]	0.00 [0.00 0.50]	0.00 [0.00 0.68]	0.00 [0.00 0.00]	0.00 [0.00 0.46]
	Excess		0.00, *p* < 2.2 × 10^−16^	0.00, *p* < 2.2 × 10^−16^	0.00, *p* < 2.2 × 10^−16^	0.00, *p* < 2.2 × 10^−16^
Cost (£)		704.80 [302.90 1559.70]	293.60 [85.17 800.64]	447.20 [185.40 1069.20]	305.60 [68.10 861.30]	349.60 [141.10 873.40]
	Excess		−411.20, *p* < 2.2 × 10^−16^	−257.60, *p* < 2.2 × 10^−16^	−399.20, *p* < 2.2 × 10^−16^	−355.20, *p* < 2.2 × 10^−16^
All-cause mortality						
	1-year: n (%)	3,775 (1.3)		30,260 (2.7)	70 (0.0)	12,320 (1.1)
	2-year: n (%)	4,500 (1.6)		35,410 (3.2)	3,220 (0.3)	23,215 (2.1)

LC: long COVID; COVID-19, no LC: COVID-19, no long COVID; ED: emergency department; GP: general practitioner. All values are to two decimal places. All the p-values are from one-sided Mann-Whitney U test. The null hypothesis the healthcare utilisation in the corresponding department (GP/OP/IP/CC/ED) from the LC group is less than or equal to the healthcare utilisation from the control group.

a *N* is indicated by median and IQR.

b ‘Excess’ denotes the difference between values the matched control cohort and the long COVID study population. A negative number means the value for the long COVID cohort was higher than the matched control cohort.

c Duration is measured in days.

d Cost includes tests/investigations.

### Cost of healthcare utilisation

We established costs for healthcare utilisation types by multiplying each category (GP, OP, IP, ED, specific imaging studies) for each patient by unit costs.^20,21^ We compared cost of healthcare utilisation between LC and each control group, using Mann-Whitney U-test. Unit costs were from national tariff (2020–2021 or other year) or research publications and adjusted for inflation (Supplementary methods). To focus analyses, for OP consultations, we concentrated on high-frequency specialties for LC referral (Supplementary methods). Similarly, we restricted analyses of investigations to CT pulmonary angiogram, Transthoracic Echocardiogram, CT head and MRI brain, based on clinical expertise, high-cost and limited availability. Although critical care costs are generally reported by specific organs, information regarding organ-specific reasons for critical care admission were lacking. Therefore, we used cost at the ‘2–3 organ support’ level as proxy for average critical care cost per day (Supplementary methods). Unit cost for ED attendance was calculated as mean of unit costs across all EDs based on national tariff 2020–2021 (Table S2).

### Statistical analysis

Baseline characteristics, including age, sex, ethnicity, IMD, geographic regions, smoking status and baseline comorbidities, were described in LC and matched control groups, comparing summary statistics. Kaplan-Meier analysis was used to estimate time to all-cause mortality and all-cause hospitalisation, within two years.

For healthcare utilisation in control populations, we calculated frequency of GP consultations, OP appointments, IP admissions (general and critical care) and ED attendances per person per month and per year, comparing with the LC cohort, using Mann-Whitney U test and odds ratios. Taking each control group as a reference group, we calculated odds of the LC group using each healthcare service. These analyses did not adjust for unmatched baseline comorbidities, including Chronic Kidney Disease, Morbid Obesity and Dementia, because these diseases were not prevalent among cases and controls (less than 4%). We did not adjust for smoking status due to a large proportion (57.4%) of unknown smoking status in the LC group.

To understand patient trajectories, we followed individuals with LC until 31 January 2023, recording proportion visiting each specialty and admissions, comparing by descriptive statistics. By multiplying each healthcare use category by corresponding unit cost (adjusted for inflation), we could compare cost of healthcare utilisation. For any missing data in a certain variable/category, we assumed utilisation in that category was zero and thus missing data were imputed as zero.

Analyses were performed according to a pre-specified analysis plan published on GitHub, along with phenotyping and analysis code (https://github.com/BHFDSC/CCU049_01).

## Results

### Baseline characteristics

We included 282,080 individuals with LC (median age [IQR] 48.00 [36.1, 58.9] years, 62.4% female, White: 83.0%) with mean FU of 395 days (standard deviation 177, range: 0–1125). The most prevalent age group was 40–59 years (44.6%). There were no differences by social deprivation (most deprived: 20.5%; least deprived: 18.8%). North West (21.2%), London (14.4%) and South West (14.2%) regions had highest representation. Comorbidities were common, whether CVD (9.4%; atrial fibrillation most common: 3.9%), cardiovascular risk factors (e.g. current smoker 4.6%, diabetes mellitus 11.2%, hypertension 12.7%) or non-CVD (e.g. cancer 21.7%, depression 20.1%, COPD 12.6%) (Table 2). Matching success rates for control populations were high (Table 1). We included 1,112,370 (FU 495 days), 1,031,285 (FU 730 days), 1,118,360 (FU 780 days) and 282,080 (FU 730 days) matched individuals with COVID-19 only, no LC; pre-pandemic; contemporary non-COVID-19 and pre-LC, respectively (Table 2).

**Table 2.** Baseline characteristics in study and matched control cohorts for analysis of healthcare utilisation in long COVID.

	LC	COVID-19 only, no LC	Pre-pandemic	Contemporary non-COVID-19
*N*	282,080	1,112,370	1,031,285	1,118,360
Follow-up (days)	395 (177)	730 (6)	495 (187)	780 (70)
Mean (SD)	range: 0–1125	range: 3–730	range: 0–1096	range: 1–791
Age mean (SD)	48.3 (15.9)	48.3 (16.3)	49.0 (16.4)	48.3 (16.2)
Median (IQR)	48.0 [36.1, 58.9]	47.9 [35.9, 58.9]	49.0 [36.0, 59.0]	48.1 [35.1, 59.1]
18–19	3815 (1.4)	15,455 (1.4)	25,195 (2.4)	13,585 (1.2)
20–29	35,490 (12.6)	1,44,890 (13.0)	132,530 (12.9)	1,40,415 (12.6)
30–39	52,280 (18.5)	2,08,250 (18.7)	171,135 (16.6)	206,640 (18.5)
40–49	61,720 (21.9)	244,395 (22.0)	221,895 (21.5)	244,800 (21.9)
50–59	64,020 (22.7)	251,040 (22.6)	240,180 (23.3)	254,835 (22.8)
60–69	37,730 (13.4)	1,43,330 (12.9)	140,340 (13.6)	150,210 (13.4)
>70	27,030 (9.6)	1,05,010 (9.4)	1,00,000 (9.7)	1,07,875 (9.6)
Female	176,110 (62.4)	694,600 (62.4)	610,640 (59.2)	696,710 (62.3)
NHS regions				
North West	59,805 (21.2)	2,33,920 (21.0)	2,24,810 (21.8)	2,35,190 (21.0)
South West	40,180 (14.2)	1,59,110 (14.3)	1,39,280 (13.5)	1,59,470 (14.3)
London	40,665 (14.4)	1,61,210 (14.5)	1,53,665 (14.9)	1,61,860 (14.5)
South East	37,810 (13.4)	1,49,615 (13.5)	1,42,535 (13.8)	1,50,620 (13.5)
West Midlands	29,460 (10.4)	1,16,235 (10.5)	1,09,300 (10.6)	1,16,885 (10.5)
East of England	23,130 (8.2)	91,445 (8.2)	80,960 (7.9)	92,235 (8.2)
Yorkshire and Humber	23,960 (8.5)	94,545 (8.5)	82,560 (8.0)	95,015 (8.5)
East Midlands	14,520 (5.2)	56,945 (5.1)	51,805 (5.0)	57,390 (5.1)
North East	12,545 (4.5)	49,340 (4.4)	46,370 (4.5)	49,690 (4.4)
IMD Quantile				
1	57,800 (20.5)	2,25,950 (20.3)	2,10,965 (20.5)	2,27,385 (20.3)
2	59,420 (21.1)	2,34,265 (21.1)	2,17,950 (21.1)	2,35,760 (21.1)
3	55,990 (19.9)	2,21,375 (19.9)	2,05,080 (19.9)	2,22,425 (19.9)
4	55,935 (19.8)	2,21,065 (19.9)	2,04,375 (19.8)	2,22,455 (19.9)
5	52,935 (18.8)	2,09,715 (18.9)	1,92,915 (18.7)	2,10,335 (18.8)
Ethnicity				
White	2,33,985 (83.0)	9,25,700 (83.2)	8,56,970 (83.1)	9,29,395 (83.1)
Black, Black British	9525 (3.4)	33,480 (3.0)	34,110 (3.3)	36,175 (3.2)
Asian, Asian British	24,400 (8.7)	82,310 (7.4)	75,320 (7.3)	79,655 (7.1)
Other ethnics	6230 (2.2)	24,030 (2.2)	26,950 (2.6)	30,430 (2.7)
Mixed	5295 (1.9)	16,650 (1.5)	15,425 (1.5)	16,145 (1.4)
Unknown	2645 (0.9)	30,205 (2.7)	22,515 (2.2)	26,555 (2.4)
Smoking status				
Current smoker	12,890 (4.6)	63,625 (5.7)	1,84,400 (17.9)	2,12,510 (19.0)
Ex-smoker	31,005 (11.0)	1,25,135 (11.3)	2,43,875 (23.7)	2,54,960 (22.8)
Never smoker	76,380 (27.1)	2,96,150 (26.6)	5,02,615 (48.7)	5,52,145 (49.4)
Unknown	1,61,810 (57.4)	6,27,460 (56.4)	1,00,395 (9.7)	98,740 (8.8)
CVD	26,435 (9.4)	88,915 (8.0)	79,725 (7.7)	85,110 (7.6)
AMI	6310 (2.2)	26,290 (2.4)	12,265 (1.2)	14,578 (1.3)
Heart failure	6920 (2.5)	27,035 (2.4)	20,380 (2.0)	23,940 (2.1)
Atrial fibrillation	11,080 (3.9)	38,390 (3.5)	34,665 (3.4)	35,725 (3.2)
Stroke	4460 (1.6)	20,155 (1.8)	18,760 (1.8)	21,060 (1.9)
Cardiomyopathy	960 (0.3)	3280 (0.3)	2745 (0.3)	3385 (0.3)
Pulmonary embolism	4940 (1.8)	5870 (0.5)	3600 (0.4)	4525 (0.4)
Deep vein thrombosis	1790 (0.6)	5475 (0.5)	4365 (0.4)	5575 (0.5)
PAD	1070 (0.4)	4560 (0.4)	5955 (0.6)	6530 (0.6)
Cancer	61,075 (21.7)	2,39,355 (21.5)	1,53,550 (14.9)	2,40,230 (21.5)
Diabetes mellitus	31,715 (11.2)	1,17,775 (10.6)	1,13,490 (11.0)	1,20,900 (10.8)
Hypertension	35,805 (12.7)	1,37,480 (12.4)	1,34,110 (13.0)	1,40,540 (12.6)
CKD	9900 (3.5)	36,475 (3.3)	28,925 (2.8)	32,975 (2.9)
COPD	35,490 (12.6)	1,35,025 (12.1)	1,27,770 (12.4)	1,38,705 (12.4)
Depression	56,760 (20.1)	2,20,240 (19.8)	1,95,105 (18.9)	2,23,105 (19.9)
Morbid obesity	10,855 (3.9)	36,605 (3.3)	20,510 (2.0)	34,580 (3.1)
Dementia	2215 (0.8)	20,530 (1.9)	5910 (0.6)	8270 (0.7)

LC: long COVID; COVID-19, no LC: COVID-19, no long COVID; CVD: cardiovascular disease; AMI: acute myocardial infarction; PAD: peripheral artery disease; CKD: chronic kidney disease.

### Healthcare utilisation

**Figure 1.**
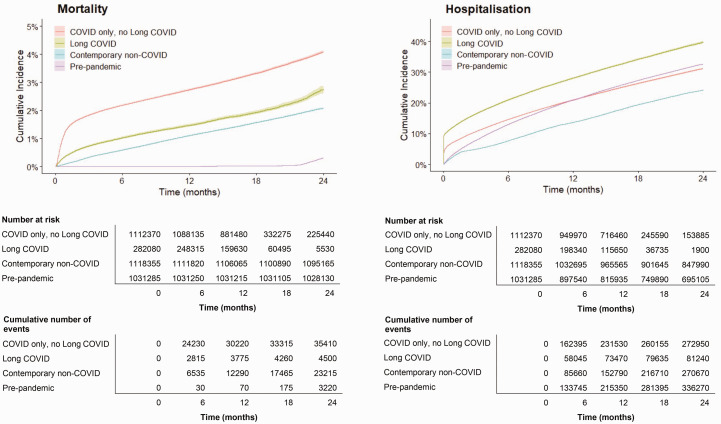
All-cause mortality and hospitalisation in individuals with long COVID compared with matched control populations.

Hospitalisation rates over two years were highest in LC and lowest in contemporary non-COVID group (Figure 1). Annually, individuals with LC had more GP consultations (median 9.90, IQR [5.47 16.99] per person per year for LC) and OP appointments (1.07, IQR [0.00 4.15] per person per year for LC) than all control groups. Although median IP admission and ED attendance for the LC group were 0(IQR [0.00 0.82]), the Mann-Whitney U test still showed that the LC group had higher ED attendance than all control groups, and significantly higher IP admissions than all control groups except the pre-pandemic group. No significant difference was found between LC and all control groups for duration of IP admission (Table 3).

**Figure 2.**
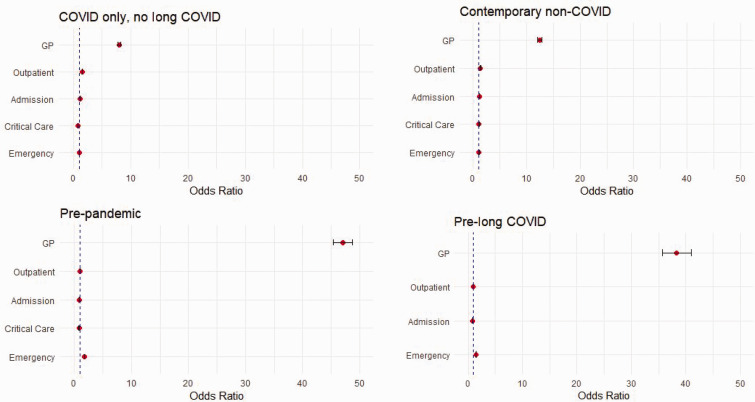
Odds ratios of healthcare utilisation in individuals with long COVID, compared with control populations.Note: We did not estimate odds for critical care utilisation in the long COVID group compared with Pre long COVID group due to zero attendance in critical care from the Pre-long COVID group (This is also shown in Table 3).

Compared with all control groups (odds = 1), odds of GP consultations were higher in the LC group (pre-LC 38.25, 95% CI [35.66, 41.03]; pre-pandemic 47.04 [45.36, 48.77]; COVID-19 only, no LC 8.02 [7.73, 8.32]; and contemporary non-COVID-19 12.41 [11.97, 12.87]) for secondary care (OP, IP admission, critical care and ED). Compared with the contemporary non-COVID-19 group, the LC group had higher odds in all four services (1.36 [1.35 1.37], 1.17 [1.16 1.17], 1.09 [1.06 1.12], 1.09 [1.08 1.10]). Compared with the COVID-19 only and no LC group, the LC group had higher odds in all secondary care, except critical care (1.53 [1.53 1.54], 1.22 [1.21 1.23], 0.80 [0.78 0.82], 1.06 [1.05 1.07], respectively). Compared with pre-pandemic, the LC group had lower or similar odds in all secondary care, except for ED (1.00 [0.99 1.00], 0.84 [0.84 0.85], 0.79 [0.77 0.81], 1.81 [1.80 1.82]). The LC group had higher odds in ED (1.44 [1.43 1.46]) but lower odds in OP (0.95 [0.94 0.96]) and IP admission (0.88 [0.87 0.89]) compared with the pre-LC group (Figure 2).

**Figure 3.**
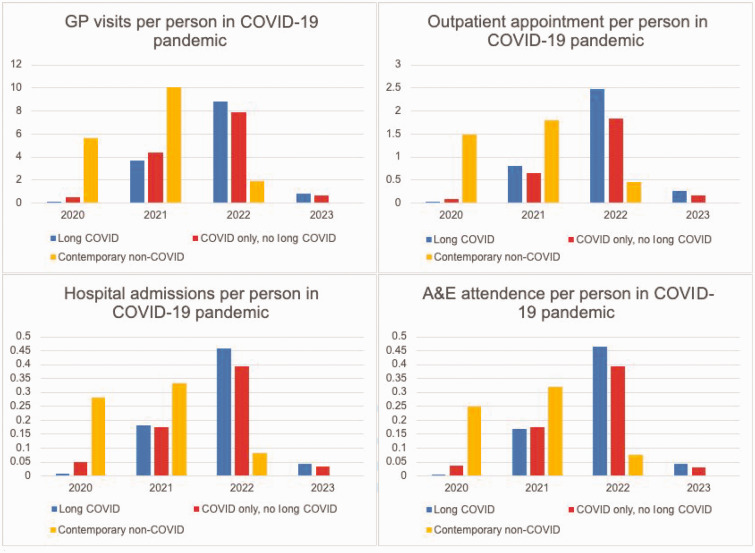
Average healthcare utilisation per year during the pandemic. Note: Contemporary non-COVID was followed from 29 January 2020 until 31 March 2022.

All healthcare utilisation categories were increased in 2022 and 2023, compared with 2020 in LC and COVID-19 only, no LC groups. In individuals with LC, number of GP consultations, OP appointments, IP admissions and ED attendances became higher than COVID-19 only, no LC group in 2022 (8.75 versus 7.89), 2021 (0.79 versus 0.65), 2021 (0.18 versus 0.17) and 2022 (0.46 versus 0.39), respectively. The contemporary non-COVID-19 group had higher utilisation in all four services than other groups in 2020 and 2021; but reduced in 2022 (note that the contemporary non-COVID-19 group was followed for full years in 2020 and 2021 but only until April 2022, when reliable testing stopped) (Figure 3).

### Cost

Median healthcare cost per person per year for individuals with LC was £704.80, compared with £293.60, £447.20, £305.60 and £349.60 in pre-LC; COVID-19 only, no LC; pre-pandemic and contemporary non-COVID-19 cohorts, respectively (Table 3).

### Care trajectory

Almost all (99.7%) people with LC used primary care post-diagnosis. Of those, 46.5% were for GP consultations, 35.5% for laboratory investigations and 18.0% for prescriptions. In the LC group, 56.6% had OP appointments post-diagnosis, with 30.3% referred from non-ED, 40.8% from GP, 3.8% from ED and 25.4% from other departments. In the LC group, 28.8% were admitted (all-cause) post-diagnosis, with 52.7% of these admissions being elective and 42.3% emergency. Overall, 1.6% of the group died (all-cause mortality) (Figure S1).

### Total burden of care over time

Total number of GP consultations, OP appointments, hospital admissions and ED attendances of the LC group over time were highest in the two months after LC diagnosis and although reduced, there was sustained utilisation over the next two years (Figure S2). There was a similar trend for number of individuals with LC and number of consultations (Figure S3).

### All-cause mortality

The COVID-19 only, no LC group had the highest mortality at one and two years (2.7% and 3.2%, respectively), compared with the LC (1.3% and 1.6%), pre-pandemic (0.0% and 0.3%) and non-COVID-19 contemporary groups (1.1% and 2.1%) (Table 3, Figure 1).

## Discussion

In the first national study of healthcare utilisation in individuals with LC to consider a range of controls, we have three main findings. First, despite low mortality rates, we showed that individuals with LC had highest healthcare utilisation over two years across GP, ED and OP care, and higher rates of hospital and critical care admission than Pre-LC group and Contemporary Non-COVID-19 groups. Second, LC care was estimated to cost over £700 (∼USD 890) per person per year, nearly 2.5 times more than care in the same individuals and age- and comorbidity-matched individuals before the pandemic, and 1.5–2 times as much as care in age- and comorbidity-matched individuals during the pandemic. Third, most LC healthcare burden was in the first two months post-diagnosis, persisting over the following two years, whether primary care, OP or IP.

We show increased healthcare utilisation in LC compared with the same individuals prior to the pandemic (thus prior to their LC), and matched individuals, pre-pandemic, therefore, discounting ‘healthcare-seeking behaviour’^22^ and baseline characteristics, respectively, as likely explanations. Moreover, we show increased healthcare utilisation in both LC and ‘COVID-19, no LC’ cohorts, compared with contemporary non-COVID-19, confirming that COVID-19 and LC are likely underlying factors. In a US study of 138,818 (mean age 60.9 years, 89% male) individuals after severe acute respiratory syndrome coronavirus 2 infection showed that at two years, LC contributed 80.4 (95% CI: 71.6–89.6) and 642.8 (596.9–689.3) disability-adjusted life years (DALYs) per 1000 persons among non-hospitalised and hospitalised individuals, where 25.3% and 21.3%, respectively, of burden was in the second year.^3^ In addition to reduced quality-of-life, we now confirm high healthcare utilisation and financial burden in a population, which is more representative by gender, age and ethnicity. Although we cannot deduce causation from our study, LC is clearly associated with multi-system effects that lead to increased healthcare utilisation, particularly in primary care, but also in all aspects of the patient pathway, with implications for resources and planning in both treatment and prevention.

Considering estimated global LC burden,^4^ there are relatively few, focused studies of healthcare utilisation and cost of care. Researchers have attempted to model or project financial burden due to COVID-19 overall, such as estimated financial burden on healthcare and pension systems in Germany of 1.7 billion euros,^15^ or DALYs^23^ or economic cost of interventions against COVID-19. Studies to-date have investigated excess cost of recovery from acute COVID-19, compared with contemporary non-COVID-19 controls,^24^ concentrating on hospitalised COVID-19,^25^ or had short-term FU rather than LC in the general population.^16^ This is the largest contemporary study of population-based individuals with confirmed LC and up to two years of FU. After initial, international focus on critical care and acute care in COVID-19, the possibility and scale of longer-term effects was known in the first wave. Our data quantify excess costs, compared with various controls, underpinning the need for prevention and management of LC, and showing that the severity, scale and cost of healthcare burden for LC are likely to far outweigh acute COVID-19 in the post-vaccination era. Therefore, pandemic preparedness and resource planning must include longer-term effects.^26^

The majority of healthcare burden is in the two months after diagnosis, probably as healthcare professionals try to rule out other diagnoses and pathologies through investigations and treatments. The early healthcare burden may represent more severe disease early on, or it may represent increased investigation load while diagnosis of LC is established. There are several longitudinal studies showing persistent symptoms and poor recovery rates over the next two years in LC, which support high healthcare usage. Most healthcare utilisation has been in primary care and ED. However, each individual had fewer appointments/admissions during the pandemic than before they got LC, which may reflect limited healthcare resources during the pandemic, or changed patient behaviour to avoid healthcare settings, or both. There are implications for patient care pathways and resource planning for LC care.

## Strengths and limitations

Our research has several strengths. We used national EHRs to study a representative LC population with validated definitions and multiple control populations to illustrate healthcare burden of LC in the most robust way to-date. The majority of the patient journey from primary care and ED to OP and IP care, including critical care, was considered in our analyses, to capture comprehensive healthcare utilisation associated with LC. We also investigated cost, trajectory and mortality. However, there are limitations. First, we are likely to have under-estimated true LC burden, partly due to under-use and under-diagnosis of LC.^19^ Second, we are likely to have underestimated duration of FU of LC because the 20 SNOMED-CT codes related to LC are not all diagnostic codes: they include both referral and assessment codes. Third, COVID-19 testing after April 2022 was not widely available, probably leading to under-testing and under-diagnosis of COVID-19 and LC. Therefore, the contemporary non-COVID-19 control group is not necessarily representative of the later pandemic. Fourth, due to low sample size by each ethnic minority group, we were only able to use two categories (‘white’ and ‘non-white’) for ethnicity as matching variables. Fifth, individuals in the pre-LC group may not be in the same situation as when they develop LC, such as baseline risk factors. Sixth, it is likely that healthcare burden and cost are sustained beyond two years, which requires further research and surveillance with longer-term FU. Seventh, data were not available for all relevant utilisation (including telephone consultations, informal healthcare and social care utilisation), which will lead to underestimation of LC healthcare utilisation. Finally, we were unable to distinguish individuals accessing dedicated post COVID-19 services which future research must focus on.

## Conclusions

Over two years, people with LC have high healthcare utilisation from primary care and ED to hospital OP and IP care with high cost to health systems. The financial, infrastructural and human resource required to meet healthcare needs requires immediate action, particularly in already over-stretched health systems. In order to avoid this cost to individuals, populations and health systems, treatment and prevention of LC must be prioritised in research, practice and policy.

## Supplemental Material

sj-pdf-1-jrs-10.1177_01410768241288345 - Supplemental material for Healthcare utilisation of 282,080 individuals with long COVID over two years: a multiple matched control, longitudinal cohort analysis Supplemental material, sj-pdf-1-jrs-10.1177_01410768241288345 for Healthcare utilisation of 282,080 individuals with long COVID over two years: a multiple matched control, longitudinal cohort analysis by Yi Mu, Ashkan Dashtban, Mehrdad A Mizani, Chris Tomlinson, Mohamed Mohamed, Mark Ashworth, Mamas Mamas, Rouven Priedon, Steffen Petersen, Evan Kontopantelis, Kim Horstmanshof, Christina Pagel, Mevhibe Hocaoğlu, Kamlesh Khunti, Richard Williams, Johan Thygesen, Paula Lorgelly, Manuel Gomes, Melissa Heightman, Amitava Banerjee and on behalf of the CVD-COVID-UK/COVID-IMPACT Consortium in Journal of the Royal Society of Medicine

## Declarations

### Data sharing

The data used in this study are available in NHS England’s SDE service for England, but as restrictions apply, they are not publicly available (https://digital.nhs.uk/services/secure-data-environment-service). The CVD-COVID-UK/COVID-IMPACT programme, led by the British Heart Foundation Data Science Centre (https://bhfdatasciencecentre.org/), received approval to access data in NHS England’s SDE service for England from the Independent Group Advising on the Release of Data (IGARD) (https://digital.nhs.uk/about-nhs-digital/corporate-information-and-documents/independent-group-advising-on-the-release-of-data) via an application made in the Data Access Request Service (DARS) Online system (ref. DARS-NIC-381078-Y9C5K) (https://digital.nhs.uk/services/data-access-request-service-dars/dars-products-and-services). The CVD-COVID-UK/COVID-IMPACT Approvals & Oversight Board (https://bhfdatasciencecentre.org/areas/cvd-covid-uk-covid-impact/) subsequently granted approval to this project to access the data within NHS England’s SDE service for England. The de-identified data used in this study were made available to accredited researchers only. Those wishing to gain access to the data should contact bhfdsc@hdruk.ac.uk in the first instance.

### Guarantor

AB.

### Provenance

Not commissioned; peer reviewed by Julie Morris.
