# Supplementary material for: Healthcare utilisation of 282,080 individuals with long COVID over two years: a multiple matched control, longitudinal cohort analysis
Source: J R Soc Med. 2024 Nov 27;117(11):369–81. doi: 10.1177/01410768241288345 (PMC11603423; doi:10.1177/01410768241288345)
Supplement: sj-pdf-1-jrs-10.1177_01410768241288345 - Supplemental material for Healthcare utilisation of 282,080 individuals with long COVID over two years: a multiple matched control, longitudinal cohort analysis [file sj-pdf-1-jrs-10.1177_01410768241288345.pdf]

SUPPLEMENTARY MATERIAL

Supplementary methods
Table S1. SNOMED and ICD-10 codes to identify patients with Long COVID in the national dataset
Table S2. Unit Cost
Figure S1. Trajectory and healthcare utilisation for the long COVID study population
Figure S2. Total GP/OP/IP/ED consultations/visits over time among people with Long COVID since diagnosis
Figure S3. Number of Long COVID patients who consulted/visited GP/OP/IP/ED over time since diagnosis

SUPPLEMENTARY MATERIAL

Supplementary methods

Outpatient: In order to focus our analyses, for outpatient consultations, we concentrated on high-frequency specialties for Long COVID patient referral: Respiratory medicine service, Physiotherapy service, Cardiology service, Diagnostic imaging service, Trauma and orthopaedic service, Ophthalmology service, Gynaecology service, Ear nose and throat service, Rheumatology service, Dermatology service, Gastroenterology service, Urology service, Clinical haematology service, Clinical haematology service, Neurology service, General surgery service, Midwifery service, Obstetrics service, General internal medicine service, Respiratory physiology service, Pain management service, and the treatment specialty code for Post COVID syndrome service (348) from 1 April 2021 (although there was no corresponding cost).

Outpatient (OP) and inpatient (IP): Due to the large amount of tests and procedures conducted for OP and IP patients, we only investigated the most common in those with Long COVID: for both OP and IP, CTPA, TTE, CT head, MRI brain; for IP only, 'fibreoptic endoscopic examination of upper gastrointestinal tract and biopsy of lesion of upper gastrointestinal tract', 'unspecified diagnostic fibreoptic endoscopic examination of upper gastrointestinal tract', 'diagnostic fibreoptic endoscopic examination of colon and biopsy of lesion of colon', 'unspecified diagnostic endoscopic examination of colon'.

Table S1. SNOMED and ICD-10 codes to identify patients with Long COVID in the national dataset

Code	Term	Source
1325161000000102	Post-COVID-19 syndrome	15 SNOMED codes(21)
1325181000000106	Ongoing symptomatic disease caused by severe acute respiratory syndrome coronavirus 2	
1325021000000106	Signposting to Your COVID Recovery	
1325031000000108	Referral to post-COVID assessment clinic	
1325041000000104	Referral to Your COVID Recovery rehabilitation platform	
1325051000000101	Newcastle post-COVID syndrome Follow-up Screening Questionnaire	
1325061000000103	Assessment using Newcastle post-COVID syndrome Follow-up Screening Questionnaire	
1325071000000105	COVID-19 Yorkshire Rehabilitation Screening tool	
1325081000000107	Assessment using COVID-19 Yorkshire Rehabilitation Screening tool	
1325091000000109	Post-COVID-19 Functional Status Scale patient self-report	
1325101000000101	Assessment using Post-COVID-19 Functional Status Scale patient self-report	
1325121000000105	Post-COVID-19 Functional Status Scale patient self-report final scale grade	
1325131000000107	Post-COVID-19 Functional Status Scale structured interview final scale grade	
1325141000000103	Assessment using Post-COVID-19 Functional Status Scale structured interview	
1325151000000100	Post-COVID-19 Functional Status Scale structured interview	
1119303003	Post-acute COVID-19 (disorder)	More codes from NHS SNOMED search engine
1325831000000100	Post-COVID-19 syndrome service (qualifier value)	
1119304009	Chronic post-COVID-19 syndrome (disorder)	
1326351000000108	Post-COVID-19 syndrome resolved (finding)	
1326321000000103	Post-COVID assessment service simple reference set (foundation metadata concept)	
U074	Post COVID-19 condition	ICD-10

Table S2. Unit Cost

Category	Cost (£) (before / after inflation)	Source of Cost	Year of the cost	Inflation rate[2]
**GP consultations (non-prescription)[1]**	33 / 34	Unit cost of health and social care staff	2020/21	1.0272
**Outpatient appointments**				
340 - Respiratory medicine service	209 / 215	national tariff 2020-2021	2020/21	1.0272
650 - Physiotherapy service	119 / 122	national tariff 2020-2021	2020/21	1.0272
320 - Cardiology service	191 / 196	national tariff 2020-2021	2020/21	1.0272
812 - Diagnostic imaging service	50 / 51	national tariff 2020-2021	2020/21	1.0272
110 - Trauma and orthopaedic service	187 / 192	national tariff 2020-2021	2020/21	1.0272
130 - Ophthalmology service	168 / 173	national tariff 2020-2021	2020/21	1.0272
502 - Gynaecology service	205 / 211	national tariff 2020-2021	2020/21	1.0272
120 - Ear nose and throat service	172 / 177	national tariff 2020-2021	2020/21	1.0272
410 - Rheumatology service	175 / 180	national tariff 2020-2021	2020/21	1.0272
330 - Dermatology service	168 / 173	national tariff 2020-2021	2020/21	1.0272
301 - Gastroenterology service	165 / 169	national tariff 2020-2021	2020/21	1.0272
101 - Urology service	144 / 148	national tariff 2020-2021	2020/21	1.0272
303 - Clinical haematology service	193 / 198	national tariff 2020-2021	2020/21	1.0272
400 - Neurology service	206 / 212	national tariff 2020-2021	2020/21	1.0272
100 - General surgery service	180 / 185	national tariff 2020-2021	2020/21	1.0272
560 - Midwifery service	118 / 121	national tariff 2020-2021	2020/21	1.0272
501 - Obstetrics service	186 / 191	national tariff 2020-2021	2020/21	1.0272
300 - General internal medicine service	211 / 217	national tariff 2020-2021	2020/21	1.0272
341 - Respiratory physiology service	159 / 163	national tariff 2020-2021	2020/21	1.0272
191 - Pain management service	238 / 244	national tariff 2020-2021	2020/21	1.0272
CTPA (U35.4)	120 / 136	(31)	2015	1.1294
TTE (U20.1)	79 / 90	(32)	2014	1.1338
CT head (U05.1)[3]	99 / 102	national tariff 2020-2021	2020/21	1.0272
MRI Brain (U05.2)[4]	176 / 181	national tariff 2020-2021	2020/21	1.0272

**Hospital admissions**				
**Days in hospital (general ward)**	587 / 650	(23)	2016/17	1.1066
CTPA (U35.4)	120 / 136	(31)	2015	1.1294
TTE (U20.1)	79 / 90	(32)	2014	1.1338
G45.1[5]	197 / 215	(33)	NHS reference costs 2017/18	1.0929
G45.9[6]	277 / 303	(33)	NHS reference costs 2017/18	1.0929
H22.1[7]	277 / 303	(33)	NHS reference costs 2017/18	1.0929
H22.9[8]	206 / 225	(33)	NHS reference costs 2017/18	1.0929
CT head (U05.1)	99 / 102	national tariff 2020-2021	2020/21	1.0272
MRI Brain (U05.2)	176 / 181	national tariff 2020-2021	2020/21	1.0272
**Days in critical care (2-3 organs support)[9]**	2552 / 2621	National tariff 2020-2021	NHS reference costs 2020/21	1.0272
**ED attendance**	269 / 276	Mean of all ED categories	NHS reference costs 2020/21	1.0272

[1] Cost per surgery lasting 9.22 minutes – GP (including direct care).

[2]Source of inflation: The NHS cost inflation index, from Unit Costs of Health and Social Care 2022 Manual https://kar.kent.ac.uk/100519/1/Unit%20Costs%20of%20health%20and%20Social%20Care%202022%20%28amended%2013%20July%202023%29.pdf

[3]We used the cost for 'Computerised Tomography Scan of One Area, without Contrast, 19 years and over'.

[4]We used the cost for 'Magnetic Resonance Imaging Scan of One Area, without Contrast, 19 years and over'.

[5]OPCS code for Fibreoptic endoscopic examination of upper gastrointestinal tract and biopsy of lesion of upper gastrointestinal tract.

[6]OPCS code for unspecified diagnostic fibreoptic endoscopic examination of upper gastrointestinal tract.

[7]OPCS code for diagnostic fibreoptic endoscopic examination of colon and biopsy of lesion of colon.

[8]OPCS code for unspecified diagnostic endoscopic examination of colon.

[9]In HES CC data, we could only observe the maximum number of organs supported, and not the duration of maximum number of organ support. Thus, we used cost for 2-3 organ support as the proxy for average cost in CC. We averaged over 'Adult Critical Care, 2 Organs Supported' (service code – CCU01; currency code - XC05Z, national average unit cost - £2,491.23) and 'Adult Critical Care, 3 Organs Supported' (service code – CCU01; currency code - XC04Z, National average unit cost - £2,612.99) in 'Non-specific, general adult critical care patients predominate'.

Table S3. Overlap between control groups

Control A	Control B	Overlap %
Pre-LC	Pre-pandemic	1.3[2]
Pre-LC	Contemporary non-COVID	0[1]
Pre-LC	COVID only, no LC	0[1]
COVID only, no LC	Pre-pandemic	6.3[2]
COVID only, no LC	Contemporary non-COVID	0[1]
Pre-pandemic	Contemporary non-COVID	8.4[2]

[1] Mutually exclusive groups were with 0% overlap.
2 Non-mutually exclusive groups were with small overlap ranging 1%-8%.

Figure S1. Trajectory and healthcare utilisation for the long COVID study population

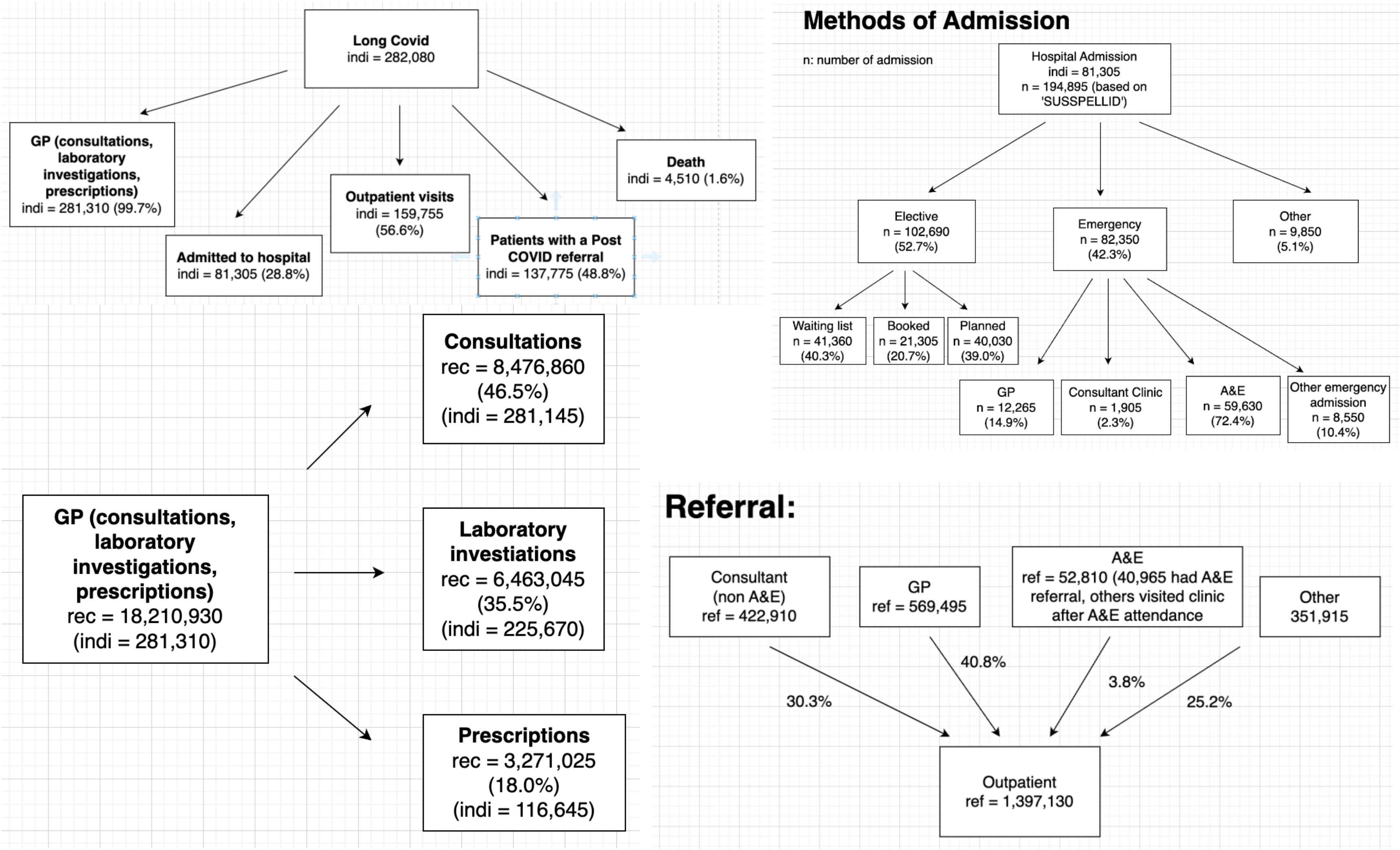

GP: General Practice; A&E is equivalent to Emergency Department

Figure S2. Total GP/OP/IP/ED consultations/visits over time among people with Long COVID since diagnosis

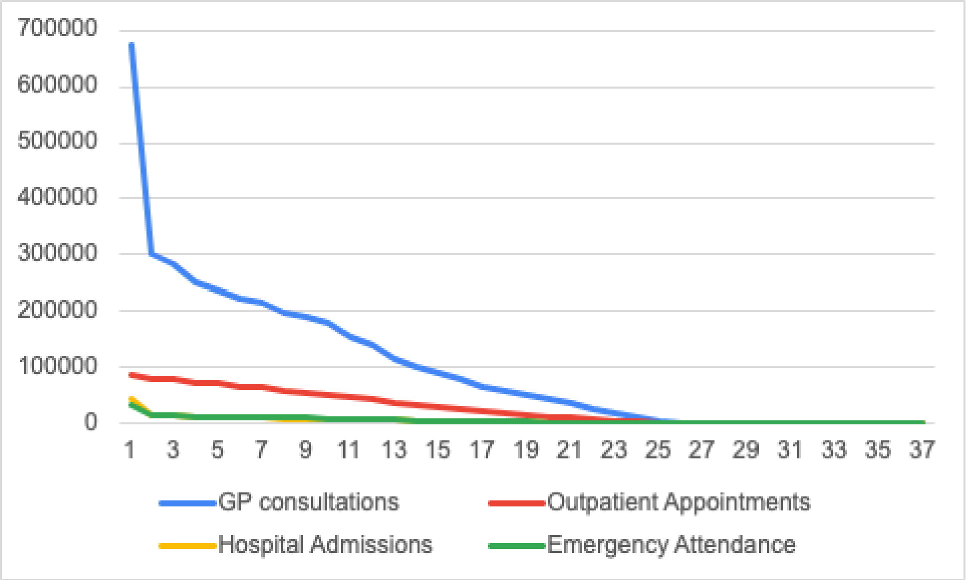

GP: General Practice; OP: Outpatient; IP: Inpatient; ED: Emergency Department

Figure S3. Number of Long COVID patients who consulted / visited GP/OP/IP/ED over time since diagnosis

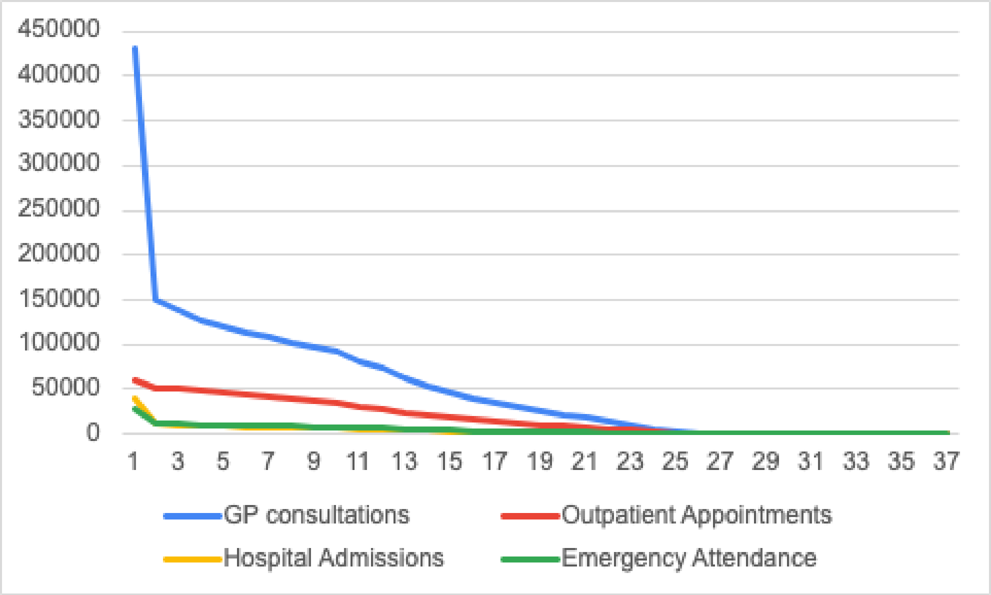

GP: General Practice; OP: Outpatient; IP: Inpatient; ED: Emergency Department
